# Explainable AI in Scene Understanding for Autonomous Vehicles in Unstructured Traffic Environments on Indian Roads Using the Inception U-Net Model with Grad-CAM Visualization

**DOI:** 10.3390/s22249677

**Published:** 2022-12-10

**Authors:** Suresh Kolekar, Shilpa Gite, Biswajeet Pradhan, Abdullah Alamri

**Affiliations:** 1 Symbiosis Centre of Applied AI (SCAAI), Symbiosis International (Deemed) University, Pune 412115, India; 2 Artificial Intelligence & Machine Learning Department, Symbiosis Institute of Technology, Symbiosis International (Deemed) University, Pune 412115, India; 3 Centre for Advanced Modelling and Geospatial Information Systems (CAMGIS), School of Civil and Environmental Engineering, Faculty of Engineering and IT, University of Technology Sydney, Ultimo, NSW 2007, Australia; 4 Earth Observation Center, Institute of Climate Change, Universiti Kebangsaan Malaysia, Bangi 43600, Selangor, Malaysia; 5 Department of Geology and Geophysics, College of Science, King Saud University, Riyadh 12371, Saudi Arabia

**Keywords:** intelligent driving, scene understanding, U-Net, inception U-Net, Grad-CAM, explainable AI

## Abstract

The intelligent transportation system, especially autonomous vehicles, has seen a lot of interest among researchers owing to the tremendous work in modern artificial intelligence (AI) techniques, especially deep neural learning. As a result of increased road accidents over the last few decades, significant industries are moving to design and develop autonomous vehicles. Understanding the surrounding environment is essential for understanding the behavior of nearby vehicles to enable the safe navigation of autonomous vehicles in crowded traffic environments. Several datasets are available for autonomous vehicles focusing only on structured driving environments. To develop an intelligent vehicle that drives in real-world traffic environments, which are unstructured by nature, there should be an availability of a dataset for an autonomous vehicle that focuses on unstructured traffic environments. Indian Driving Lite dataset (IDD-Lite), focused on an unstructured driving environment, was released as an online competition in NCPPRIPG 2019. This study proposed an explainable inception-based U-Net model with Grad-CAM visualization for semantic segmentation that combines an inception-based module as an encoder for automatic extraction of features and passes to a decoder for the reconstruction of the segmentation feature map. The black-box nature of deep neural networks failed to build trust within consumers. Grad-CAM is used to interpret the deep-learning-based inception U-Net model to increase consumer trust. The proposed inception U-net with Grad-CAM model achieves 0.622 intersection over union (IoU) on the Indian Driving Dataset (IDD-Lite), outperforming the state-of-the-art (SOTA) deep neural-network-based segmentation models.

## 1. Introduction

The World Health Organization (WHO) survey states that roughly 1.3 million deaths are caused yearly because of road accidents [1]. An intelligent transportation system (ITS) is the solution to deal with road traffic. The intelligent transportation system has seen a lot of interest among researchers because of advancements in modern machine learning techniques, especially deep learning [2]. An autonomous vehicle is the most essential and critical part of an intelligent transportation system [3]. The autonomous vehicle comprises three significant sub-tasks, namely, vehicle detection [4], behavior prediction of nearby vehicles [5], and vehicle control [6]. Understanding the surrounding environment is essential for understanding the behavior of nearby vehicles and pedestrians to enable the safe navigation of autonomous vehicles in crowded traffic environments. Segmentation is the critical phase for scene understanding of the surrounding environment [7]. Semantic segmentation for scene understanding is the pre- or post-processing step of various computer vision tasks, especially in an autonomous vehicle. Semantic segmentation labels each image pixel to a particular class, having the potential in the field of intelligent driving [8].

Despite several ongoing research works, traffic scene understanding in unstructured environments, especially for an autonomous vehicle, is a very complex task compared with human-level performance [9]. Before deep learning models, semantic segmentations were based on manually hand-crafted features. Owing to recent advancements in convolutional neural networks, it is used as a backbone in image classification tasks by reducing image resolution to obtain a high-level feature map [10]. As CNN reduces the input image into a tiny feature map, spatial information is lost and affects the overall performance of the semantic segmentation task [11]. This study proposed an inception-based U-Net model for semantic segmentation to deal with this problem. The inception module is used as an encoder for extracting the feature map, and this feature map is passed to a decoder to reconstruct a segmented image.

Various popular autonomous vehicle datasets are available in the literature, like KITTI [12], LYFT [13], Apolloscape [14], and Argoverse [15], which assume a structured environment like in developed countries. However, such an environment is not present for driving in developing countries, especially India. Generally, traffic on Indian roads is highly unstructured and unpredictable [16]. Indian Driving Dataset (IDD) is the first unstructured and unpredictable driving scenarios dataset launched by NCVPRIPG 2019.

This paper focuses on accurately segmenting objects like drivable or non-drivable areas, vehicles, human beings, and roadside objects from images taken from unstructured traffic roads using the inception U-Net model. It is challenging to accurately segment images from dense traffic because of unstructured roads and unpredictable driving patterns, especially on Indian roads. The rationale behind using the inception U-Net model for semantic segmentation is the hybrid combination of two widely used deep learning architectures, namely, inception architecture by Google and U-Net architecture. The proposed architecture replaced the default convolutional layer with the inception layer of GooLe-Net [17].

The proposed inception U-Net model is a black-box by nature and is utilized to semantically segment images from unstructured Indian roadways for an autonomous vehicle. It is challenging to build trust commercially in an autonomous vehicle among consumers owing to its black-box nature [18]. Explainable artificial intelligence (XAI) techniques are used to interpret the results obtained from black-box deep learning models. Grad-CAM is the post-hoc explainability technique primarily used in convolutional neural-network-based models [19]. The results obtained from semantic segmentation using the inception U-Net model are passed to Grad-CAM for post-hoc explainability to build consumer trust.

The main contributions of this work can be summed up as follows:

- To propose the inception-based U-Net Model for semantic segmentation of images taken from unstructured and unpredicted traffic roads;
- To assess and analyse the performance of the inception-based U-Net model with state-of-the-art (SOTA) deep neural-network-based semantic segmentation models;
- To interpret the results obtained from the black-box inception-based U-Net model using the Grad-CAM post-hoc explainability technique.

Semantic segmentation of images using deep neural networks is based on a U-shape-based encoder–decoder architecture. The novelty of this research work is to replace the convolutional layer with an inception layer to improve the performance of the proposed model. The inception module uses a multi-scale convolutional layer applied independently on the input image at every stage using a different filter size, which are then concatenated and passed to the next layer. As the human visual cortex identifies patterns at different scales, the inception module benefits the U-Net model by extracting features from different scales. Unfortunately, different researchers employed various deep learning techniques on autonomous vehicles that are black-box in nature. Therefore, in this work, to fill the aforementioned research gap, an explainable artificial intelligence (XAI) technique is used to interpret the results obtained by the inception U-Net model using Grad-CAM post-hoc explainability techniques. The findings of this study can be used as a guideline for developing autonomous vehicles using the interpretable AI model for developing countries.

The remainder of the paper is structured as follows. Section 2 discusses relevant research in the domains of autonomous vehicles and semantic segmentation. The proposed architecture is seen in Section 3. Section 4 describes the Indian Driving Dataset Lite (IDD-Lite) dataset and the experimental setup. Section 5 presents the experimental results. Section 6 summarizes the findings, and Section 7 concludes the study and gives future directions.

## 2. Related Work

Scene understanding is an important task to understand the behavior of surrounding vehicles and pedestrians for safe and secure navigation of intelligent driving [20]. Image segmentation is the essential phase for scene understanding of the surrounding environment. Semantic segmentation for scene understanding is the pre- or post-processing step of various computer vision tasks [8]. Before the deep neural network evolution, classical or traditional methods were used for semantic segmentation. Classical methods were mainly focused on hand-crafted features like the histogram of gradient (HoG) [21] methods. These features are passed to classifiers like the naïve Bayes, the support vector machine, and random forest. These methods depend on hand-crafted features instead of understanding the data structure to perform pixel-level classification for semantic segmentation [22].

After the evolution of deep learning techniques, especially convolutional neural network like VGG [23], ResNet [24], Xception [25], and recently GooLe-Net [26], pixel-level classification for semantic segmentation using U-shaped architectures such as VGG16-UNet and ResNet18-UNet has achieved state-of-the-art results. Audebert et al., in 2017, proposed the VGG16-UNet model for semantic segmentation for vehicle extraction. The encoder layer is based on the convolutional layers of the VGG-16 model. CNN’s overall accuracy improved by 10% when using the VGG16 pre-trained network at the cost of a greater inference time [27]. Popular semantic segmentation architectures are encoder- and decoder-based systems. The encoder comprises several blocks, each of which accepts an input picture or feature map and creates a series of down-sampled feature maps that progressively detects higher-level features. The decoder network mirrors the encoder network and gradually up-samples the encoder network’s output. Individual decoder blocks are linked to corresponding encoder blocks by skip links to aid in the recovery of fine-grained features lost during down-sampling. Transposed convolutions with learnable weights are commonly used for up-sampling. VGG16 was used for feature extraction and applied to the decoder model to reconstruct a segmentation map. VGG16 feature extractor outperforms ResNet and E-Net. An ensemble of different feature extractors like VGG16, ResNet, and E-net could be used to improve the performance of segmentation model [28]. While driving on structured traffic roads, it is important to follow traffic rules for safety of autonomous vehicles. However, such a structured traffic road environment is very useful to reduce the complexity of the autonomous vehicle navigation system. However, in unstructured traffic roads, there is unavailability of concrete traffic rules and absence of lane information [29]. It is a very difficult task to train an autonomous vehicle in unstructured environments owing to the large amount of complexity involved. The success of autonomous vehicles in developing countries is largely dependent on AI algorithms that consider the unstructured nature of traffic.

Advancements in artificial intelligence algorithms help to solve various critical issues in autonomous vehicles like object detection, behavior understanding of nearby vehicles, and vehicle control [30]. The performance of deep learning models is mainly dependent on a large amount of annotated datasets. However, collecting large amounts of real-world datasets for an autonomous vehicle is time-consuming and cost-ineffective. Many research groups published open access autonomous vehicle datasets that assume a structured environment like in developed countries to deal with the issue. However, such an environment is not present for driving in developing countries, especially India. Generally, traffic on Indian roads is highly unstructured and unpredictable [31]. Indian Driving Dataset (IDD) is the first unstructured driving scenarios dataset launched by NCVPRIPG 2019.

Modified U-Net (Mod-UNet) was presented by Tiwari et al. [31] as a unique segmentation model for effective vehicle segmentation into images of road traffic with crowded and unstructured traffic patterns. The suggested model is based on the U-Net architectural family and combines low-level and higher-level feature maps. For semantic segmentation, the U-Net deep learning model is a well-known method. It is divided into three stages: contraction, bottleneck, and expansion. Mod-UNet achieved IoU scores of 0.61 and 0.82 on IDD-Lite and autorickshaw dataset, respectively, at the cost of a greater inference time compared with U-Net. Baheti et al. [32] proposed the EfficientNet-UNet model for semantic segmentation of the IDDLite dataset. EfficientNet is combined with the U-Net model as an encoder for extracting high-level features and a decoder for reconstructing feature maps for segmentation. Initially, a new baseline architecture called EfficientNetB0 was built, and it was scaled up to generate a family of EffcientNets using a compound scaling mechanism. This method has resulted in eight EfficientNets versions, notably EfficientNetB0 through EfficientNetB7. U-Net with EfficientNetB7 encoder achieved a greater IoU score compared with the remaining seven EfficientsNets.

Porzi et al. [33] proposed a unique segmentation head that mixes multi-scale features generated by a feature pyramid network (FPN) with contextual information provided by a lightweight DeepLab-like module in real time. This seamless scene segmentation applied to three challenging datasets, i.e., Indian Driving dataset (IDD), Mapillary vistas, and Citiscape. The proposed architecture provides a unique CNN architecture for obtaining seamless scene segmentation results, consisting of semantic segmentation and instance segmentation modules operating on top of a single network backbone. The performance of seamless scene segmentation is better compared with individual models consisting of semantic and instance segmentation with the cost of fractional computation time. Singh et al. [34] analyzed four object detection models, three semantic segmentation models, and three instance segmentation models on three datasets, namely, Cityscape, BDD, and IDD. Object detection models perform worse on IDD compared with Cityscape and BDD owing to the unstructured nature of the IDD dataset. Instance segmentation and semantic segmentation perform better on Cityscape and IDD compared with BDD owing to the complexity of the BDD dataset. DeepLab3+ with a dilated residual network was proposed by Baheti et al. [35] for semantic segmentation of the Indian Driving Dataset (IDD). It improves feature map resolution by replacing down sampling layers with dilated convolutions. Dilated residual networks can segment small objects while maintaining neuronal spatial accuracy, leading to improved segmentation performance.

The deep-learning-based encoder and decoder model used for semantic segmentation of images from unstructured Indian roads for autonomous vehicles is black-box by nature [36]. It is challenging to build trust commercially in an autonomous vehicle among consumers because of its black-box nature. Explainable artificial intelligence (XAI) techniques are used to interpret the results obtained from black-box deep learning models [37]. Grad-CAM is the post-hoc explainability technique primarily used in the convolutional-neural-network-based model. Gradient-weighted class activation mapping (Grad-CAM) employs gradients from any target idea to construct a crude localization map emphasizing key regions in the picture for concept prediction. Grad-CAM may be used with a broad variety of CNN model families.

According to the literature, unstructured traffic datasets for autonomous vehicles are the least researched dataset. At the time of writing, no experiments have been conducted to interpret the results of an AI model applied to an unstructured traffic dataset using the GradCAM post-hoc explainability model. This research proposed an inception-based U-net model with Grad-CAM for interpreting semantic segmentation of images taken from unstructured traffic roads. The inception module is used as an encoder for extracting the feature map, and this feature map is passed to the decoder to reconstruct the segmented image. Grad-CAM is used to visualize the results obtained from the employed models in order to interpret and explain the results.

## 3. Proposed Methods

Inception U-Net with Grad-CAM is proposed for semantic segmentation of input images captured on unstructured Indian roads for autonomous vehicles.

### 3.1. Inception U-Net Architecture

**Figure 1:**
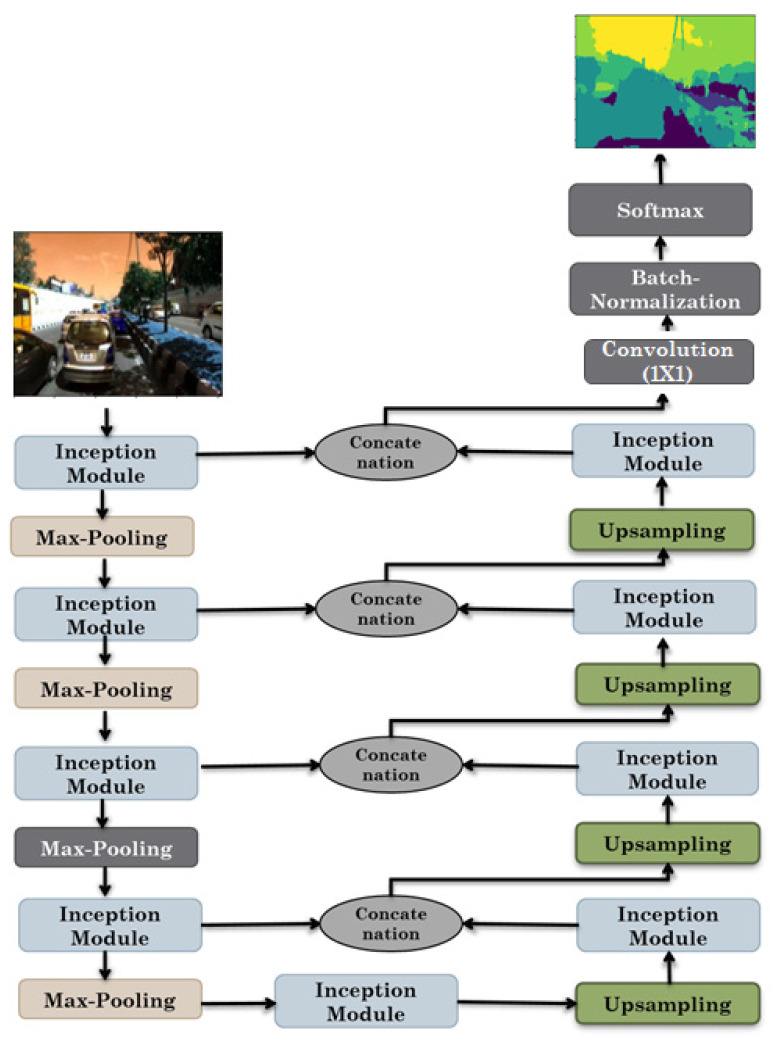
Schematic design of the proposed model.

The proposed inception U-Net model is a hybrid model combining two state-of-the-art (SOTA) deep learning models, the inception architecture of Google and U-Net architecture. Inception layers of GoogLe-Net replace the convolutional layers in the U-Net model. The detailed architecture of Inception-UNet is presented in Figure 1. Using the inception module, the proposed model used a contracting and expanding architecture proposed in U-Net architecture with a bottleneck in the middle. Each layer on the contracting side consists of the inception layer, followed by max-pooling. On the other hand, each layer on the expanding side consists of the concatenation of the inception module and features from a corresponding layer of the contracting side, followed by up-sampling [38].

The number of filters increased by double at each layer on the contracting side and reduced by half at each layer on expanding side. The height and width of the input and output images are the same. At the output of the expanding side, the convolutional layer is used, followed by the softmax activation function, to perform pixel-level classification to obtain a binary segmentation image as output [38].

### 3.2. Inception Module

**Figure 2:**
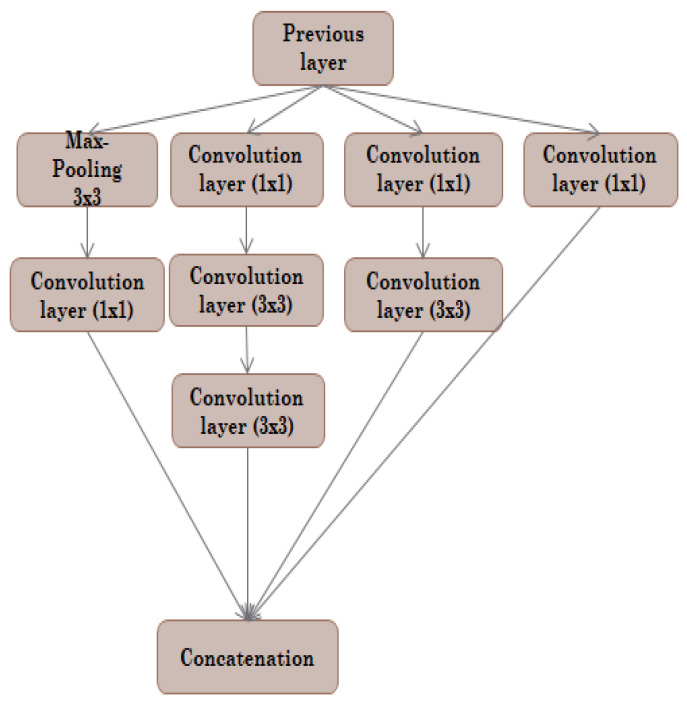
Inception module with convolution.

The inception module used filters of different sizes at the same level to make the network broader instead of deeper. The inception module is illustrated in Figure 2. The naïve inception module performs convolution on input with three filters (1 × 1, 3 × 3, 5 × 5). Max-pooling was performed additionally. The results of each are concatenated and sent to the next stage. An extra 1 × 1 convolution is added before the 3 × 3 convolution to make the inception module with a reduced dimension. Moreover, 1 × 1 convolution is added before 5 × 5 convolution, and 3 × 3 convolution is added after 5 × 5 convolution. Lastly, 5 × 5 convolution is replaced by 3 × 3 convolution. The inception module achieves state-of-the-art results with this multi-scale model training [39].

The rationale behind using an inception module in the U-Net segmentation model is to approximate an optimal local sparse structure in convolution layers. The inception module allows the use of multiple filter sizes for a single image block, which then concatenates and passes to the next layer to extract meaningful features from the input image block.

### 3.3. Gradient-Weighted Class Activation Map (Grad-CAM)

Gradient-weighted class activation map (Grad-CAM) creates a heat map of the input image that highlights the essential parts of an image by utilizing the gradients of the final convolutional layer’s target. It takes the feature maps from the final layer and weights each channel by the gradient of the class concerning the channel. It reflects how strongly the input image activates specific channels based on their importance concerning the class. There is no need to retrain the model or change the current architecture [40]. However, IDDLite is a multiclass segmentation image dataset where each image consists of multiple objects that need to be segmented. There are seven classes: drivable, non-drivable, living things, vehicles, roadside objects, and far objects. GradCAM is used as post-hoc explainability to visualize heat map of final convolutional layer for each class.

## 4. Experimental Setup

This section discusses the Indian Driving Dataset (IDD-Lite), the training process of the inception U-Net model, and the performance measures used to evaluate the performance of our model.

### 4.1. Dataset

**Figure 3:**
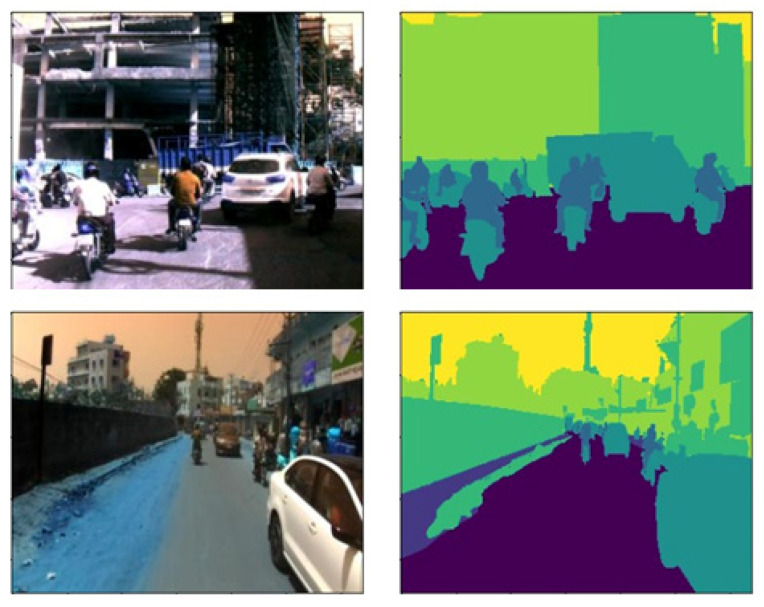
Original images with ground truth.

The results of the proposed architecture were evaluated using an IDDLite dataset. IDD-Lite, a semantic segmentation dataset on unstructured and unpredictable driving situations, is provided by the Indian Institute of Information Technology (IIIT) in Hyderabad, India. IDD-Lite, a lite version of the same dataset with the same level of statistics as IDD, has been provided for the resource-constrained scenario. The IDD-Lite dataset includes 1404 training samples, 204 validation samples, and 408 testing samples that depict realistic Indian driving scenarios like complex obstructions, fuzzy road boundaries, a diverse range of vehicles and pedestrians, varying lighting conditions, and a disregard for traffic rules. The dataset is divided into seven categories: driving, non-driving, live beings, cars, roadside items, distance objects, and sky. Figure 3 illustrates various representative samples from this collection, as well as their corresponding ground truths.

### 4.2. Model Training

In the model training, each image from IDD-Lite is first resized to 128 × 256. Input dimensions for model training are 128 × 256 × 3, where 3 represents the RGB channels, and output dimensions are 128 × 256 × 8, where 8 represents the classes. The IDD-Lite dataset includes 1404 training samples and 204 validation samples. The inception U-Net model was trained for 50 epochs, and one epoch took approximately 1 h and 20 min. The learning rate of the proposed model training was 0.001. Next, we used the Adam optimizer to optimize categorical cross-entropy loss functions. The segmentation model is implemented by Keras, and an experiment was conducted on NVidia K80 GPU. Table 1 shows the performance measures on training samples.

### 4.3. Performance Measures

Accuracy, specificity, sensitivity, F-score, and intersection over union are the five metrics used to evaluate the results of semantic segmentation models [31]. Each performance metric is explored further below.

- Accuracy: It is the ratio of addition of true positives (TP) and true negatives (TN) with addition of true positives (TP), true negatives (TN), false positives (FP), and false negatives (FN). The formula of accuracy is presented in (1).

(1) $$\mathrm{Accuracy}=\frac{\left(\mathrm{TP}+\mathrm{TN}\right)}{\left(\mathrm{TP}+\mathrm{FP}+\mathrm{FN}+\mathrm{TN}\right)}$$

- Sensitivity: It is the ratio of true positives and the summation of false negatives and true positives. The formula of sensitivity is presented in (2).

(2) $$\mathrm{Sensitivity}=\frac{\mathrm{TP}}{\left(\mathrm{FN}+\mathrm{TP}\right)}$$

- Specificity: It is the ratio of true negatives (TN) and the addition of true negatives (TN) and false positives (FP). The formula of specificity is presented in (3).

(3) $$\mathrm{Specificity}=\frac{\mathrm{TN}}{\left(\mathrm{TN}+\mathrm{FP}\right)}$$

- F-score: It is the harmonic mean of recall and precision. The formula of F-score is presented in (4).

(4) $$\mathrm{F-Score}=\frac{2\times \mathrm{TN}}{\left(2\times \mathrm{TN}+\mathrm{FP}+\mathrm{FN}\right)}$$

- Intersection over union (IoU): It is the average overlap between the predicted and ground truth divided by the union area between the predicted and ground truth. The formula of IoU is presented in (5).

(5) $$\mathrm{IoU score}=\frac{\mathrm{Area}\mathrm{of}\mathrm{overlap}}{\mathrm{Area}\mathrm{of}\mathrm{union}}$$

## 5. Results

**Figure 4:**
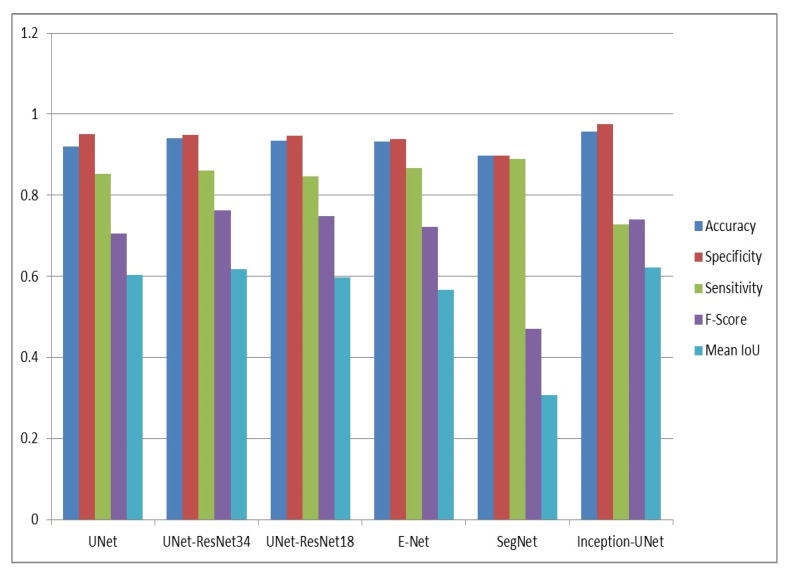
Comparative analysis of the proposed inception U-Net model with the state-of-the-art models.

The proposed inception U-Net model was evaluated through experiments to validate the results. The results of the inception U-Net model are compared with state-of-the-art models, namely, U-Net, UNet-ResNet18, UNet-ResNet34, SegNet, and E-Net. Performance evaluation of the proposed inception U-Net model and state-of-the-art models based on accuracy, specificity, sensitivity, f-score, and intersection over union is illustrated in Table 2 and Table 3, respectively. Table 4 illustrates a comparative analysis of the proposed model with state-of-the-art-models (SOTA) based on mIoU. Graphical comparative analysis of the proposed inception U-Net model with state-of-the-art models, namely UNet, UNet-ResNet18, UNet-ResNet34, SegNet, and E-Net, is presented in Figure 4. Class-wise performance of inception U-Net based on intersection over union on the IDD-Lite dataset is illustrated in Table 5.

The IoU, accuracy, specificity, sensitivity, and F-score of the proposed inception U-Net model are 0.622, 0.958, 0.975, 0.728, and 0.740, respectively. The IoU metric assesses the performance of semantic segmentation models. The mIoUscore of inception U-Net is 0.622, which is greater than the state-of-the-art segmentation models.

**Figure 5:**
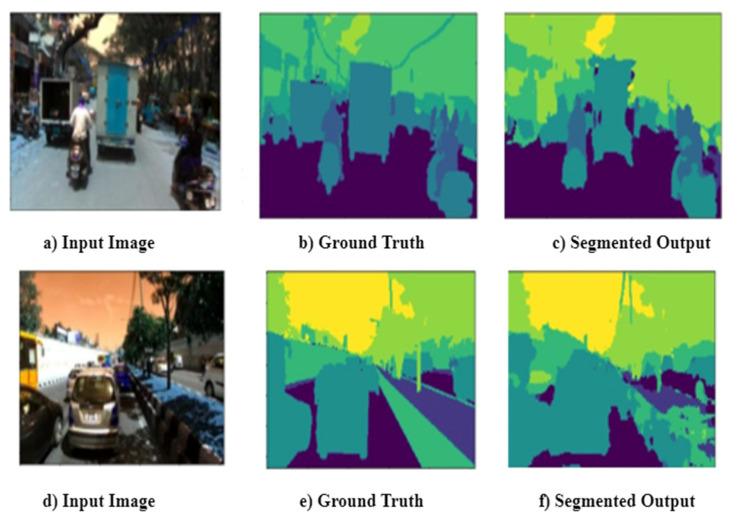
Inception U-Net predicted output with: (**a**) input image; (**b**) ground truth; (**c**) segmented output; (**d**) input image; (**e**) ground truth; and (**f**) segmented output.

**Figure 6:**
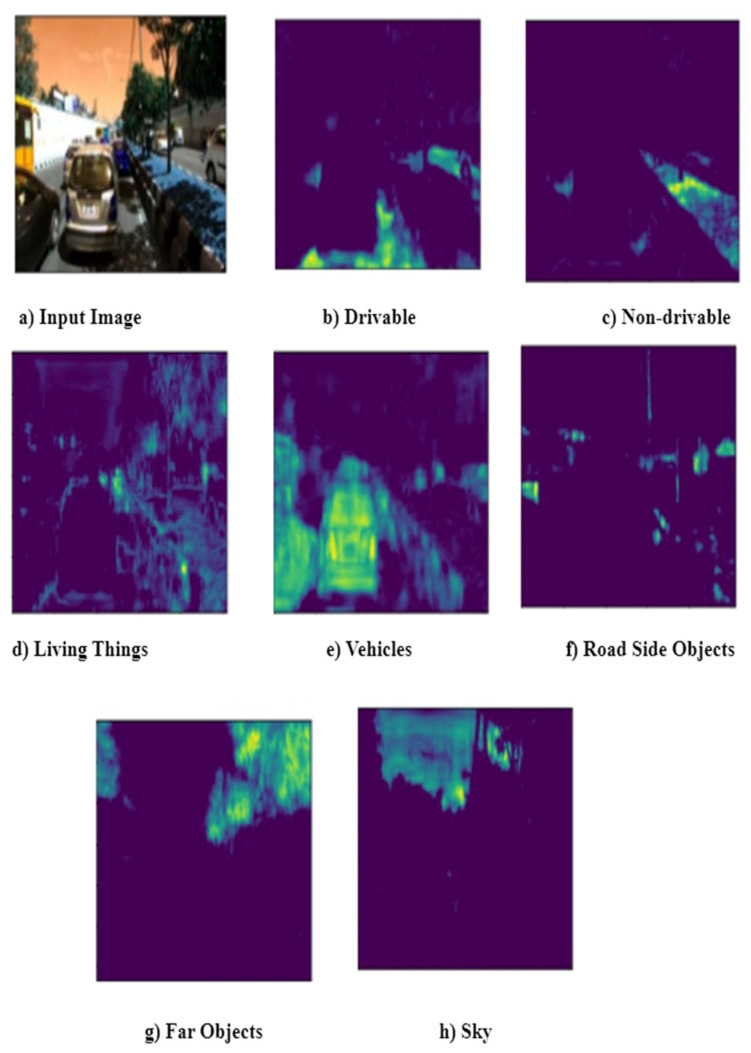
Class-wise final convolutional layer output of inception U-Net with Grad-CAM model for: (**a**) input image; (**b**) drivable; (**c**) non-drivable; (**d**) living things: (**e**) vehicles; (**f**) road side objects; (**g**) far objects; and (**h**) sky.

Grad-CAM is the post-hoc explainability technique used to interpret the results obtained from the inception U-Net Model. Grad-CAM creates a heat map of the input image that highlights an image’s essential parts by utilizing the final convolutional gradients. It takes the feature maps from the final layer and weights each channel by the gradient of the class concerning the channel. Figure 5 illustrates the sample input image and segmented output obtained from the inception U-Net model. The same sample image was passed through Grad-CAM post-hoc explainability techniques and created the final convolutional output with a heat map for each class. There are seven classes: drivable, non-drivable, living things, vehicles, roadside objects, and far objects. Figure 6 illustrates the final convolution output with a heatmap for each class.

## 6. Discussions

Semantic segmentation of traffic images is essential for scene understanding to understand the behavior of nearby vehicles and pedestrians for safe navigation of the autonomous vehicle. Industries like LYFT, WYMO, and Argoverse release publicly available datasets to help researchers to work on them. These datasets are collected in developed countries where there are structured traffic roads. Traffic in developing countries like India is unstructured and unpredictable. IDD released a dataset collected on Indian unstructured and unpredictable roads.

This research applied inception U-Net on the IDD-Lite dataset for semantic segmentation and passed predicted results through Grad-CAM for interpreting results. The inception U-Net model was compared with ResNet-UNet models, E-Net, and DRN_ResNet models. The performance measures of the inception U-Net model on the training dataset are listed in the table.

The IoU score and pixel-wise accuracy of the proposed model using the training dataset are 0.798 and 0.983, respectively, which is better compared with the state-of-the-art (SOTA) models. The performance evaluation of the proposed models and state-of-the-art models on the validation dataset is illustrated in Table 2 and Table 3, respectively. The IoU is 0.622 and pixel-wise accuracy is 0.958 for the validation dataset. It is observed that the inception U-Net model has a better IoU and accuracy compared with the state-of-the-art models, namely, U-Net, UNet-ResNet34, UNet-ResNet18, E-Net, and SegNet. Table 4 compares the proposed model with the SOTA models using the IDD-Lite validation dataset and mIoU. The proposed model was evaluated against several SOTA models built on IoU, including UNet-ResNet34, UNet-ResNet18, DRN-ResNet18, DRN-ResNet50, and ERF Net. The proposed model outperforms the SOTA U-shaped semantic segmentation models in terms of the IoU score. In the U-Net model, residual networks serve as the framework for the ResNet-UNet model. However, the DRN-ResNet-UNet model employs a dilated residual network, an enhanced residual network, as the backbone. Additionally, the convolution layer in the U-Net model is replaced with a multi scale inception model in the proposed model.

Class-wise intersection over the union of inception U-Net is presented in Table 5. It is observed that the intersection over the union is better for drivable areas than other classes. This drivable area is the region of interest for safe, autonomous vehicle navigation. The sample input image and corresponding segmented output, along with ground truth, are presented in Figure 5. Class-wise final layer output passed through Grad-CAM is presented in Figure 6. Gram-CAM takes the feature maps from the final layer and weights each channel by the gradient of the class concerning the channel. It reflects how strongly the input image activates specific channels based on their importance concerning the class. Figure 6b,c show the results of Grad-CAM for the drivable class and non-drivable class. It is observed that the region of interest (ROI) for class drivable is segmented accurately. This drivable region is very important to consider for safe navigation of autonomous vehicles in dense traffic. Figure 6d,e show the results of Grad-CAM for the living things class and vehicles class, respectively. Accurate segmentation of living things and vehicles in the nearby scene is very important to avoid accident. Figure 6f,g show the results of Grad-CAM for road-side object class and far distant object class, respectively. This region of road-side object and far distant object is very important for long-term planning of autonomous vehicle navigation.

The IoU of the the inception U-Net model followed by those of UNet-ResNet34, U-Net, UNet-ResNet18, E-Net, and SegNet are 0.622, 0.6174, 0.6031, 0.5981, 0.566, and 0.3076, respectively. It is observed that the inception U-Net model outperforms the state-of-the-art (SOTA) U-shaped encoder–decoder segmentation models based on IoU. The performance of semantic segmentation models is assessed by accuracy, sensitivity, specificity, and f-score in addition to intersection over union (IoU), as it involves pixel-level classification. The accuracy of the inception U-Net model followed by those of UNet-ResNet34, UNet-ResNet18, E-Net, U-Net, and SegNet are 0.958, 0.9398, 0.9356, 0.9321, 0.9203, and 0.8971, respectively. In terms of accuracy and specificity, inception U-Net outperforms the state-of-the-art (SOTA) U-shaped encoder–decoder segmentation models. However, in terms of sensitivity and F-score, SegNet and UNet-ResNet34 surpass other the segmentation models, respectively.

The inception U-Net model is not computationally effective as the number of trainable parameters is in the millions. Training time and inference time are more due to a huge amount of trainable parameters. During training of the inception U-Net model, it focuses on relevant as well as non-relevant activations that lead to wastage of computational resources.

## 7. Conclusions and Future Directions

Behavior understanding of surrounding road side objects is very important for the design and development of an autonomous vehicle. In the literature, the semantic segmentation technique is generally used to segment images from surrounding traffic scenes to understand the behavior of roadside objects. Previous work revealed that the implementation of semantic segmentation models based on U-Net architecture was encouraging. However, deep learning models are black boxes, so it can be difficult to communicate their results to the end users. Therefore, in this work, for the first time, XAI is used to understand the results of deep learning models applied to segment traffic scene captured on unstructured traffic roads.

In this paper, inception U-Net with Grad-CAM is used for semantic segmentation of unstructured and unpredictable IDD-Lite datasets. Intersection over union (IoU) is an important performance measure to evaluate the performance of segmentation models. The inception U-Net model achieves 0.622 intersections over union (IoU). The results are passed through Grad-CAM to explain the inception U-Net model’s results. It is observed that the inception U-Net model’s performance is better compared with the state-of-the-art segmentation models. The design and development of autonomous vehicles employing XAI has become critical to commercial success. Interpretation of the proposed inception U-Net model using Grad-CAM will help autonomous vehicles in achieving commercial success. In the future, the attention-based inception U-Net model can be used to make it computationally effective. The attention-based inception U-Net model only focuses on relevant activations, while training avoids non-relevant activation. The attention-based inception U-Net avoids wastage of computation resource and makes it computationally effective.

## Figures and Tables

**Table 1:** Performance of inception U-Net on the IDD-Lite training dataset.

Model	Accuracy	Specificity	Sensitivity	F-Score	Mean IoU
Inception U-Net	0.983	0.990	0.870	0.879	0.798

**Table 2:** Performance evaluation of inception U-Net on the IDD-Lite validation dataset.

Model	Accuracy	Specificity	Sensitivity	F-Score	Mean IoU
Inception U-Net	0.958	0.975	0.728	0.740	0.622

**Table 3:** Performance evaluation of state-of-the-art-models (SOTA) on the IDD-Lite validation dataset [31].

Model	Accuracy	Specificity	Sensitivity	F-Score	Mean IoU
U-Net	0.9203	0.9500	0.8534	0.7056	0.6031
UNet-ResNet34	0.9398	0.9484	0.8617	0.7635	0.6174
UNet-ResNet18	0.9356	0.9469	0.8472	0.7485	0.5981
E-Net	0.9321	0.9395	0.8669	0.7229	0.566
SegNet	0.8971	0.8975	0.8896	0.4705	0.3076

**Table 4:** Comparative analysis of the proposed model with state-of-the-art-models (SOTA) on the IDD-Lite validation dataset based on mIoU.

Model	Proposed Model	DRN Res Net 50 [37]	UNet Res Net 34 [31]	U Net [31]	UNet Res Net 18 [31]	DRN Res Net 18 [37]	E-Net [31]	ERF Net [37]
mIoU	0.622	0.618	0.617	0.603	0.598	0.585	0.566	0.554

**Table 5:** Class-wise performance of inception U-Net based on IoU on the IDD-Lite validation dataset.

C1: Drivable	C2: Non- Drivable	C3: Living Things	C4: Vehicles	C5: Roadside Objects	C6: Far Objects	C7: Sky	mIoU
0.923	0.333	0.371	0.664	0.404	0.712	0.950	0.622

## Data Availability

The data used in this study are available at https://idd.insaan.iiit.ac.in/dataset/details/, accessed on 10 September 2022.
